# Construction of Ontology Augmented Networks for Protein Complex Prediction

**DOI:** 10.1371/journal.pone.0062077

**Published:** 2013-05-01

**Authors:** Yijia Zhang, Hongfei Lin, Zhihao Yang, Jian Wang

**Affiliations:** College of Computer Science and Technology, Dalian University of Technology, Dalian, Liaoning, China; University of Pittsburgh, United States of America

## Abstract

Protein complexes are of great importance in understanding the principles of cellular organization and function. The increase in available protein-protein interaction data, gene ontology and other resources make it possible to develop computational methods for protein complex prediction. Most existing methods focus mainly on the topological structure of protein-protein interaction networks, and largely ignore the gene ontology annotation information. In this article, we constructed ontology augmented networks with protein-protein interaction data and gene ontology, which effectively unified the topological structure of protein-protein interaction networks and the similarity of gene ontology annotations into unified distance measures. After constructing ontology augmented networks, a novel method (clustering based on ontology augmented networks) was proposed to predict protein complexes, which was capable of taking into account the topological structure of the protein-protein interaction network, as well as the similarity of gene ontology annotations. Our method was applied to two different yeast protein-protein interaction datasets and predicted many well-known complexes. The experimental results showed that (i) ontology augmented networks and the unified distance measure can effectively combine the structure closeness and gene ontology annotation similarity; (ii) our method is valuable in predicting protein complexes and has higher F1 and accuracy compared to other competing methods.

## Introduction

Protein complexes are groups of two or more associated polypeptide chains, which play a critical role in many biological processes. Many proteins are functional only after they are assembled into a protein complex and interact with other proteins in this complex. Even in the relatively simple model organism *Saccharomyces cerevisiae*, these complexes are comprised of many subunits that work in a coherent fashion. Therefore, protein complexes are important molecular entities in cellular organization, and are of great importance in unveiling the secrets of cellular organization and function.

As protein complexes are groups of proteins that interact with each other, they are generally dense subgraphs in protein-protein interaction (PPI) networks [1], [2]. The increase in available PPI data makes it possible to predict protein complexes in PPI networks. Several computational methods for protein complex prediction typically focus on the extraction of dense regions in the PPI networks based on graph theory, including MCL [3], MCODE [4], LCMA [5], CFinder [6] and PCP [7]. However, these methods ignore the biological properties of protein complexes. In general, the proteins in a complex have similar biological properties, but PPI networks cannot provide such vital information. In addition, PPI data produced by high-throughput experiments are often associated with high false positive and false negative rates [8], [9].

To address these problems, other valuable resources are gradually being used for protein complex prediction. For example, several recent studies [10], [11] have investigated gene expression data to improve protein complex prediction. These studies mainly defined specific scoring methods based on gene expression data, and constructed more reliable weighted PPI networks. The intuition behind them is that the weighted PPI networks should better represent the actual interaction network than the initial binary PPI networks.

**Figure 1:**
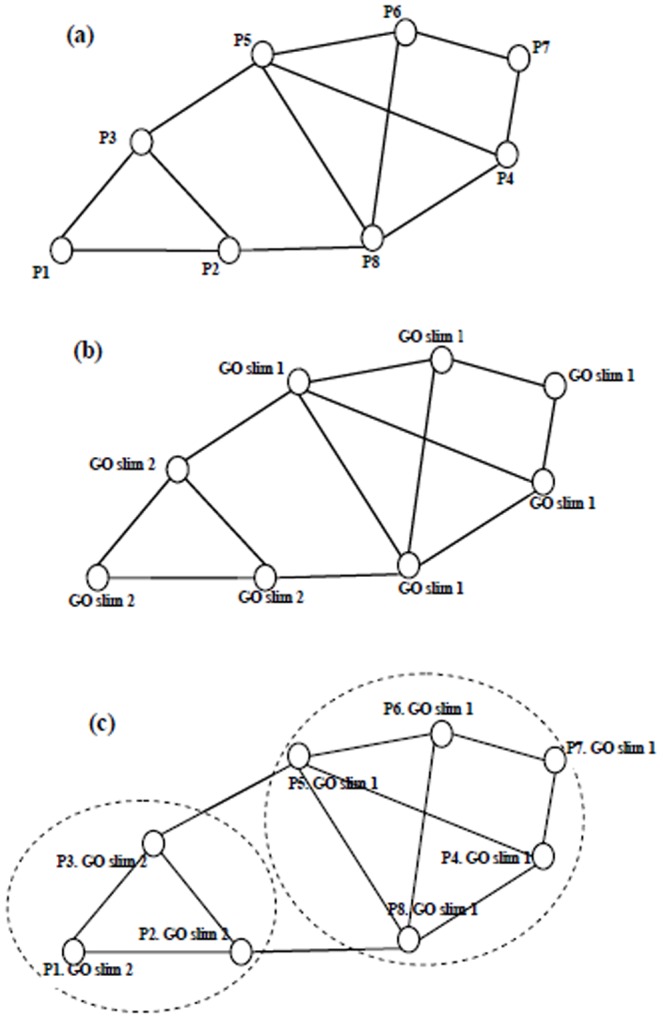
An example of protein complex prediction: (a) A PPI network is constructed by eight proteins. (b) The PPI network is annotated by GO slims. (c) Prediction of two protein complexes in the PPI network based on structural and GO annotation similarities.

Gene Ontology (GO) is another useful resource, and is currently one of the most comprehensive ontology databases in the bioinformatics community [12]. GO aims to standardize the annotation of genes and gene products across species and provides a controlled vocabulary of terms for describing gene product biological properties, which is a significant addition to PPI data for protein complex prediction. Due to the inherent biological properties of protein complexes, the ideal method for protein complex prediction should generate clusters in PPI networks which have a cohesive topological structure with similar GO annotations, by balancing the topological structure and GO annotation similarities. Figure 1 shows an example of protein complex prediction. Figure 1 (a) is a simple PPI network where a vertex represents a protein and an edge represents the interaction between two proteins. Figure 1 (b) is the PPI network annotated by GO slims. As we can see, due to the presence of noise and the complex connectivity of PPI data, it is hard to predict protein complexes from the PPI network in Figure 1 (a). However, if we consider the GO annotation information of each protein in Figure 1 (b), we can predict two complexes reasonably well in Figure 1 (c).

In this study we determined how to predict protein complexes based on both the topological structure of PPI networks and GO annotation similarities. We proposed a novel method for protein complex prediction, called COAN, based on attribute graph clustering theory [13]. The key to our method was to integrate the PPI data and GO into a unified framework by constructing ontology augmented networks. In the ontology augmented networks, we used a unified distance measure to estimate the pairwise vertex closeness. Based on the ontology augmented graph and unified distance measure, COAN generated seed cliques from the maximal cliques in the PPI networks, and expanded clusters starting from the seed cliques. In the experimental section, we showed that COAN was competitive or superior in performance, compared with the state-of-the-art methods used for protein complex prediction.

## Materials and Methods

### Ontology augmented networks

Some resources, such as gene expression data, have been used to assess the reliability of protein interactions. These methods usually assign a score to each protein pair. Unlike these methods, we integrated the PPI data and GO into a unified framework by constructing ontology augmented networks, based on attribute graph clustering theory [13].

The GO database is currently one of the most comprehensive and well-curated ontology databases in the bioinformatics community. GO provides GO terms to describe gene product characteristics in the following three different aspects, (I) biological process referring to a biological objective to which the gene or gene product contributes; (II) molecular function defined as the biochemical activity of a gene product; (III) cellular component referring to the place in the cell where a gene product is active. GO slims are cut-down versions of the GO ontologies containing a subset of GO terms. Compared with GO terms, GO slims give a broad overview of the ontology content without the detail of the specific fine-grained terms. GO slims are particularly useful for giving a summary of the results of GO annotation of a genome, microarray, or cDNA collection when broad classification of gene product function is required. The studies [14], [15] have showed the proteins in a protein complex generally share one or more GO term annotations. Since GO slims give a broader overview of ontology content than GO terms, we used GO slims to annotate PPI data in this study. Next, we introduce how to construct ontology augmented networks.

**Figure 2:**
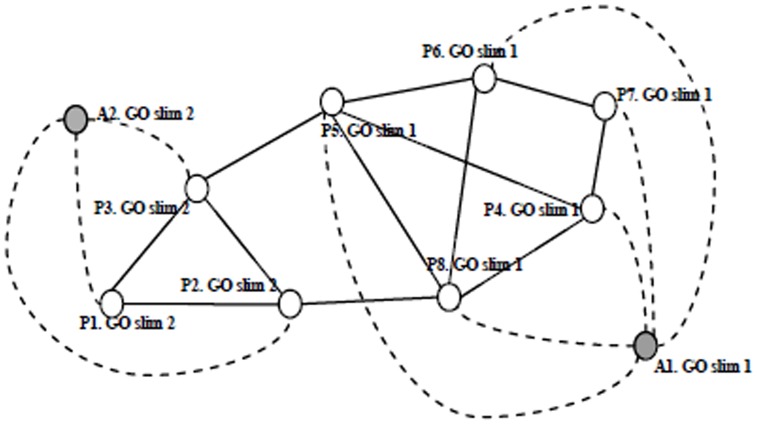
Ontology augmented graph with GO slims.

Given a PPI network 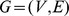 and the GO slim annotations set 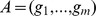, each protein could be annotated by one or more GO slims in 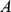. For 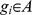, we add a “dummy” vertex 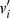 in 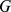. An ontology augmented network is denoted as 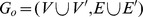 where 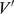 is the set of GO “dummy” vertices. An edge 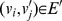 denotes the protein 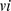 is annotated by GO slim 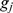. An edge 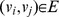 is called a PPI edge and an edge 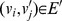 is called a GO annotation edge. Figure 2 is the ontology augmented network for the example in Figure 1. Two GO “dummy” vertices “A1. GO slim 1” and “A2. GO slim 2” are added. Proteins with corresponding GO annotations are connected to the two “dummy” vertices, respectively, in the dash line.

### Unified distance measure

**Figure 3:**
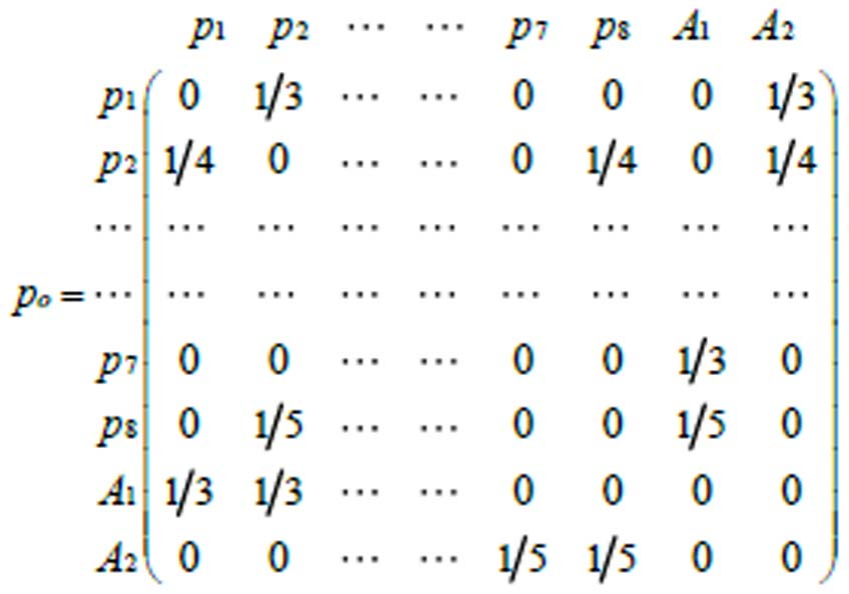
Transition probability matrix of the ontology augmented network example.

The transition matrix 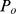 of the ontology augmented network is a 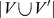 by 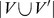 matrix. The transition probability is defined as follows: The transition probability from protein 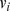 to its neighbor 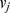 through a PPI edge or a GO annotation edge is 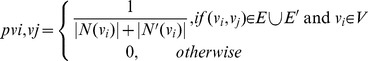
(1)where 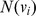 represents the set of proteins directly connecting with protein 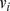 in the ontology augmented network, and 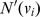 represents the set of dummy vertices, namely GO slim annotations, directly connecting with protein 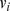. The transition probability from GO annotation 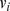 to protein 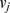 through a GO annotation edge is
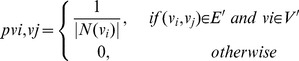
(2)Since there is no edge between two GO annotations, the transition probability between 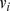 and 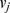 is 0.



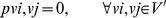
(3)Combining Equations (1)-(3), the transition probability matrix 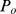 of an ontology augmented network 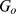 can be calculated. Figure 3 is a transition probability matrix for our example in Figure 2.

When the transition matrix 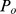 is multiplied by itself, each 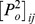 contains the summed transition probability of paths from protein 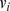 to protein 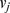 through one intervening vertex, that is, the length of paths is two. Similarly, for any length 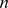, the summed transition probability from protein 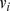 to protein 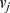 can be determined by calculating 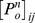. The unified distance on the ontology augmented network is defined as follows: 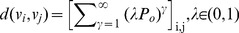
(4)


Where 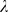 is the delay parameter. The matrix form of the unified distance is 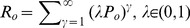
(5)Due to 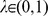, the unified distance matrix 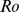 can be efficiently calculated by Equation (6). Matrix inversion is roughly of cubic time complexity.



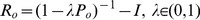
(6)


We use unified distance to measure protein pair closeness. One important difference between the unified distance on the ontology augmented network 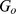 and that on the original PPI network 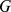 is that, if two proteins 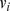 and 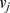 have the same GO annotation 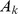, they will have a new common neighbor, thus there is a random walk path between 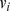 and 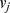 through 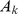. The more GO annotations two proteins share, the more random walk paths exist between the pair of proteins. The increase in paths between the pair of proteins 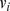 and 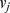 will enlarge the value of 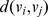. Based on ontology augmented networks, we effectively unify the topology structure of PPI networks and the similarity of GO annotations into unified distance measures.

### The COAN algorithm

The COAN algorithm broadly consists of two phases. In the first phase, COAN generates seed cliques from all the maximal cliques. Firstly, COAN ranks the cliques based on the unified distance measure. Then, COAN chooses the top rank clique as the seed clique, and removes or prunes the others. This process is repeated until the candidate clique set is empty. In the second phase, COAN expands clusters starting from the seed cliques by adding the close neighbor proteins.

As the existing PPI networks are usually sparse, enumerating all maximal cliques does not pose a problem [10]. COAN uses the cliques algorithm proposed by Tomita et al. [16] to enumerate all maximal cliques with size no less than 3 from initial PPI networks. All maximal cliques make up the candidate clique set 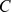. COAN uses density function to measure the closeness of each clique. The density function is defined as follows: 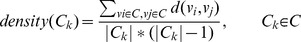
(7)where 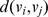 is the unified distance of two proteins 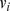 and 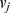 on ontology augmented networks. If the clique has a large density value, the clique generally has strong connectivity and shares more common GO annotations. Therefore, the density function takes into account both the structure connectivity of PPI networks and GO annotation similarity. In order to choose large density cliques as seed cliques, COAN ranks all the maximal cliques in descending order of their density value.

In general, the maximal cliques overlap with each other. With COAN the seed cliques do not overlap as the overlapped cliques are removed or pruned. Given a candidate clique set ranked in descending order of their density value, denoted as 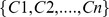, the COAN algorithm deletes the top rank clique 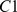 from 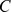 and inserts 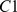 into the seed clique set 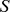. Then, the COAN algorithm removes or prunes the overlapped cliques as follows: For any other clique 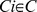, COAN checks whether 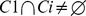. If such 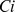 exists, COAN further checks whether 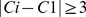 or not. If 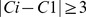, 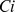 is replaced by 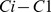, otherwise 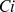 is removed directly. These steps are repeated until candidate clique set 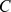 is empty. Consequently, COAN generates the seed clique set 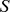, and the seed cliques are not overlapped.

**Figure 4:**
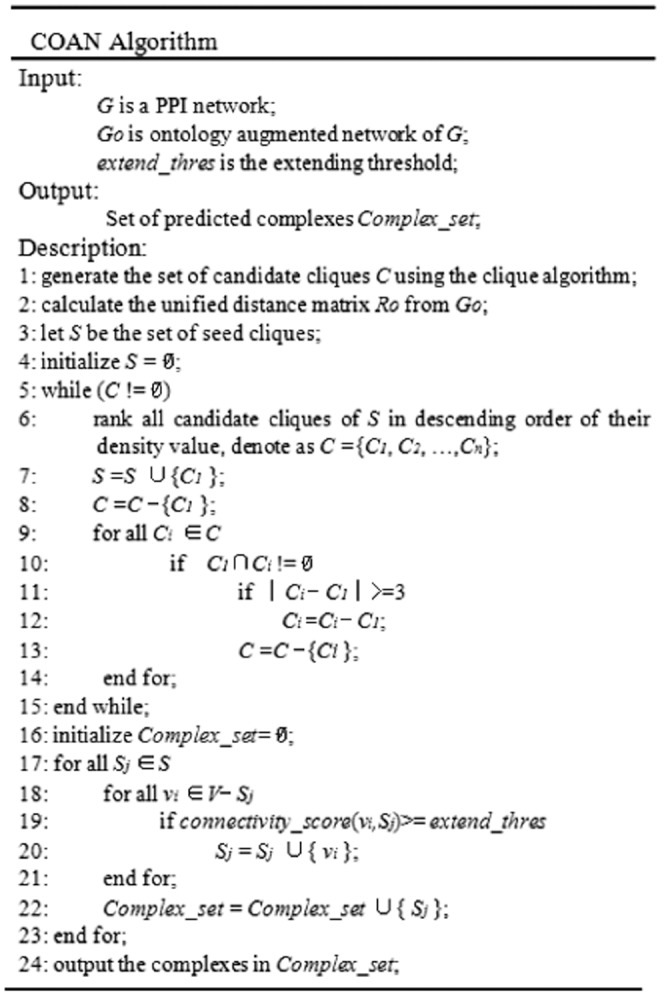
The pseudo-codes of the COAN algorithm.

In the second phase, COAN expands the seed cliques by adding the close neighbor proteins. We use the connectivity score to measure how strongly a protein 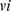 is connected to a seed clique 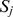, where 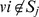. The connectivity score of 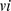 with respect to 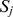 is defined as follows: 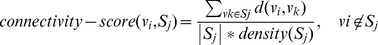
(8)If the 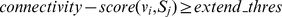, then 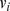 is added to 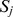. Here, 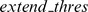 is a predefined threshold for extending. Thus the final predicted complexes will be generated by adding the close proteins to the seed cliques. Figure 4 shows the pseudo-codes of the COAN algorithm.

## Results and Discussion

In this section, we first describe the datasets and evaluation metrics used in our experiments, and then study the impact of the 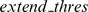 on COAN. We compared COAN with the state-of-the-art methods including CMC [10], COACH [15] and HUNTER [11]. Finally, we present some protein complexes predicted by COAN with detailed information. The Source Code S1 in Supplementary Information is the source code of COAN.

### Datasets

The two PPI datasets used were the DIP dataset [17] and Krogan dataset [18], respectively. The DIP database contains 4928 proteins and 17208 interactions, and the Krogan database contains 2675 proteins and 7080 interactions.

The reference complex dataset was CYC2008 [19] which is a comprehensive catalogue of 408 manually curated heterometric protein complexes reliably backed by small-scale experiments reported and used as benchmark complexes in most methods.

### Evaluation metrics

Overall, there are two types of evaluation metrics used to evaluate the quality of predicted complexes and compute the overall precision of the prediction methods.

One type of evaluation metrics are Precision, Recall and *F1* which are commonly used in bioinformatics and machine learning. Let 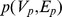 be a predicted complex and 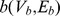 be a reference complex. The neighborhood affinity score 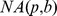 between 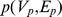 and 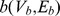 is defined as follows:
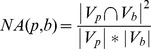
(9)


If 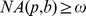, then we consider 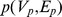 and 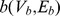 to match each other. We set 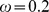 in our experiment, which is the same as most methods for protein complex prediction [4], [5], [19]-[21]. Let 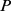 and 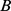 denote the sets of complexes predicted by a method and reference complex, respectively. Let 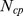 be the number of predicted complexes which match at least one reference complex and 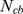 be the number of reference complexes that match at least one predicted complex. Precision, Recall and the *F1* measure are defined as follows: 
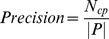
(10)

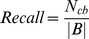
(11)

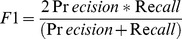
(12)


Precision measures the fidelity of the predicted complex set. Recall quantifies the extent to which a predicted complex set captures the known complexes in the reference set. The *F1* measure provides a reasonable combination of both precision and recall, which can be used to evaluate the overall performance.

Another type of evaluation metrics are sensitivity, positive predictive value (PPV) and accuracy which were recently proposed to evaluate the performance of the protein complex prediction methods [22]. The definitions of these parameters are described in detail by Xiao et al. [23].

### The effect of extend_thres

Firstly, we kept 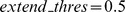 and evaluated the effect of 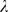 in Equations (4) on the performance of COAN by setting 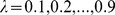, respectively. Overall, COAN achieved best performance, when 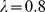. Secondly, we kept 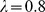 and studied the effect of 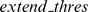 on the performance of COAN by setting 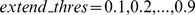, respectively. The detailed experimental results on the DIP dataset with different 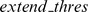 are shown in Table 1.

**Table 1:** The effect of *extend_thres* on the performance of COAN on the DIP database.

Extend _–_ thres	Size	Precision	Recall	F1	Sensitivity	PPV	Accuracy
Threshold = 0.1	118	0.274	0.174	0.213	**0.707**	0.192	0.368
Threshold = 0.2	77	0.339	0.252	0.29	0.657	0.27	0.421
Threshold = 0.3	69	0.405	0.324	0.36	0.588	0.338	0.446
Threshold = 0.4	50	0.462	0.407	0.433	0.515	0.41	0.46
Threshold = 0.5	37	0.48	0.431	0.455	0.464	0.481	0.472
Threshold = 0.6	31	**0.486**	**0.438**	**0.461**	0.435	0.555	0.491
Threshold = 0.7	28	0.457	0.412	0.433	0.403	0.598	0.491
Threshold = 0.8	21	0.441	0.404	0.422	0.383	0.636	**0.494**
Threshold = 0.9	14	0.433	0.397	0.414	0.369	**0.659**	0.493

The word ‘size’ refers to the size of the largest predicted complex with different *extend_thres*. The highest value in each row is in bold.

As shown in Table 1, the COAN algorithm is sensitive to 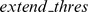. When 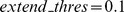, the precision and recall were only 0.274 and 0.174, respectively. This indicates that too many proteins were added to the seed cliques to construct complexes in the expanding step, because the value of 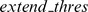 was too small. In particular, the size of the largest predicted complex with 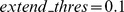 was 118, which is too large for protein complexes. With an increase in 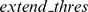, the precision and recall improved. When 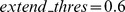, the precision and recall were highest. In addition, the highest value of *F1* was 0.461, which is generally used to evaluate overall performance. When 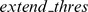 was increased from 0.6 to 0.9, the precision, recall and *F1* all decreased. When 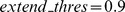, the size of the largest predicted complex was only 14. This indicated that only the closest proteins were added to the seed clique in the expanding step, however, the proteins closely connected to part of the seed clique may well be missed.

In general, high sensitivity values indicate that the prediction has good coverage of the proteins in the reference complexes, while high PPV values indicate that the predicted complexes are likely to be true positive [23]. When 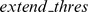 was changed from 0.1 to 0.9, the PPV always increased but sensitivity dropped sharply. This is mainly because with an increase in 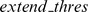, the size of predicted complexes gradually decreases and only the closest proteins can be added to the seed cliques. Therefore, the predicted complexes are more likely to be true positive or a part of the reference complexes, when 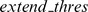 is larger. accuracy is defined as the geometric average of sensitivity and PPV. Similar to *F1*, accuracy increased when 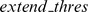 was changed from 0.1 to 0.6. However, when 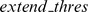 ranged from 0.6 to 0.9, accuracy did not change appreciably, and was about 0.49.

### Comparison of COAN with other methods

In this experiment, we compared COAN with the state-of-the-art methods: CMC [10], COACH [15], HUNTER [11] MCODE [4] and MCL [3]. The results using the DIP dataset and the Krogan dataset evaluated with the CYC2008 dataset are listed in Table 2 and Table 3, respectively.

**Table 2:** Performance comparison of protein complex prediction methods using the DIP dataset.

Methods	#Complexes	Size	Precision	Recall	*F1*	Sensitivity	PPV	Accuracy
COAN	383	31	0.486	0.438	**0.461**	0.435	0.555	**0.491**
COACH	730	85	0.364	**0.468**	0.41	0.544	0.38	0.455
CMC	173	49	0.595	0.287	0.387	0.399	**0.566**	0.475
HUNTER	92	160	**0.685**	0.199	0.308	0.496	0.467	0.482
MCODE	77	60	0.468	0.098	0.162	0.279	0.352	0.313
MCL	372	498	0.21	0.232	0.221	**0.555**	0.331	0.429

The ‘#Complexes’ refers to the number of predicted complexes, and ‘Size’ refers to the size of the largest predicted complex. *extend_thres* was set at 0.6 for COAN. The highest score is in bold.

**Table 3:** Performance comparison of protein complex prediction methods using the Krogan dataset.

Methods	#Complexes	Size	Precision	Recall	*F1*	Sensitivity	PPV	Accuracy
COAN	237	20	0.709	0.331	**0.451**	0.388	**0.646**	**0.501**
COACH	345	24	0.617	**0.343**	0.441	0.432	0.544	0.485
CMC	111	24	0.748	0.235	0.358	0.381	0.589	0.474
HUNTER	74	67	**0.865**	0.199	0.323	0.374	0.569	0.462
MCODE	72	52	0.75	0.159	0.263	0.27	0.552	0.386
MCL	309	486	0.291	0.245	0.266	**0.57**	0.396	0.475

The ‘#Complexes’ refers to the number of predicted complexes, and ‘‘Size’ refers to the size of the largest predicted complex. *extend_thres* was set at 0.6 for COAN. The highest score is in bold.

As shown in Table 2, COAN outperformed other methods using the DIP dataset. In particular, COAN achieved an *F1* of 0.461, which was significantly superior to the other methods. Compared to COAN, COACH predicted more complexes, which was beneficial in achieving high recall and sensitivity. In contrast, MCODE only predicted 77 complexes, which resulted in the worst recall of 0.098 and *F1* of 0.162. HUNTER predicted 92 complexes and achieved the highest precision of 0.685. MCL and CMC achieved the highest sensitivity of 0.555 and PPV of 0.566, respectively. In addition, we noticed that the size of the largest predicted complex by the four methods was very different. The largest predicted complex by MCL consisted of 498 proteins, which was far beyond the normal size protein complex.

Next, we compared the four methods using the Krogan dataset. From Table 3, it can be seen that the results using the Krogan dataset were similar to the results using the DIP dataset. COAN predicted 237 complexes, and achieved best performance in the overall evaluation metrics, *F1*, PPV and accuracy. COACH predicted 345 complexes, and achieved highest recall of 0.343. HUNTER and MCL achieved best precision 0.865 and sensitivity 0.57, respectively. MCODE only predicted 72 complexes, and achieved worst recall 0.159.

Overall, COAN predicted many protein complexes using the DIP and Krogan datasets, and outperformed other methods in the major evaluation metrics, *F1* and accuracy.

**Figure 5:**
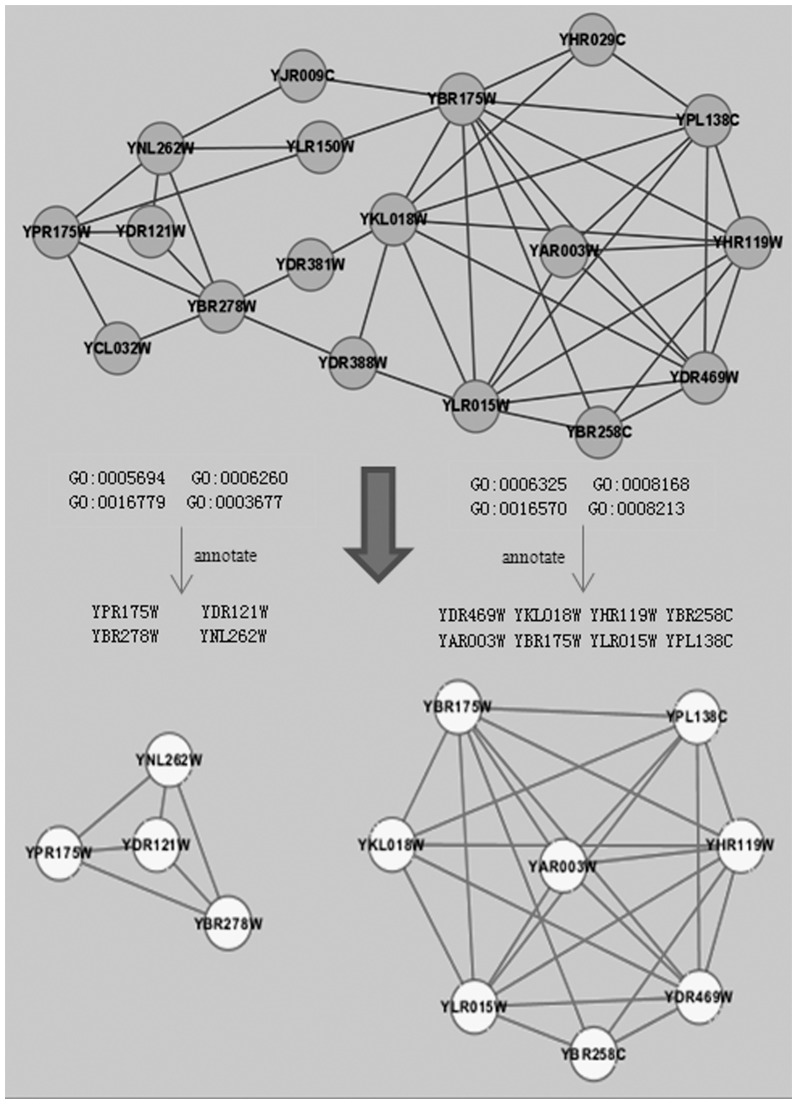
Two protein complexes predicted by COAN method on Krogan dataset.

In addition, Figure 5 gives an example of two complexes predicted by COAN on Krogan dataset. Due to the complex connectivity of PPI networks, it is difficult to accurately predict complexes only based on topology structure information of PPI networks. If the PPI network is annotated by GO slim, it can be noticed that some proteins share common GO slim annotations. For instance, “YPR175W”, “YDR121W”, “YBR278W” and “YNL262W” share common GO slim annotations “GO:0005694”, “GO:0006260”, “GO:0016779” and “GO:0003677” in Figure 5. Based on such valuable GO slim annotations information, two complexes can be predicted by COAN relatively easily.

### Examples of predicted complexes

Examples of predicted complexes using the DIP dataset are presented in Table 4 with the p-values of the three GO domains. In general, a predicted complex is considered to be statistically significant if the p-value is less than 0.01. Therefore, a smaller p-value represents a higher biological meaning in Table 4. We used the tool SGD's GO::TermFinder [24] to calculate p-value. From Table 4, it can be seen that some predicted complexes (ID1- ID6) matched the reference complex dataset well with high p-values. Other predicted complexes (ID7-ID9) were not matched with the reference dataset. However, they also had high biologically functional homogeneity and local density. Therefore, they are possible real protein complexes which are still undiscovered by biologists. These results provide clues for biologists to verify and identify new protein complexes.

**Table 4:** Examples of predicted complexes using the DIP dataset.

ID	Predicted complexes	NA	GO biological processes		GO molecular functions		GO cellular components	
			Annotation	P-value	Annotation	P-value	Annotation	P-value
1	YDR469W YKL018W YHR119W YBR258C YAR003W YBR175W YLR015W YPL138C	1	GO:0051568 (histone H3-K4 methylation)	1.21e-20	GO:0042800 (histone methylase activity)	7.56e-26	GO:0035097 (histone methyltransferase complex)	1.68e-25
2	YBR234C YNR035C YKL013C YIL062C YDL029W YLR370C YJR065C	1	GO:0030041 (actin filament polymerization)	3.99e-18	GO:0003779 (actin binding)	3.66e-12	GO:0005885 (Arp2/3 protein complex)	1.16e-21
3	YHR187W YMR312W YGR200C YPL101W YPL086C YLR384C	1	GO:0006400 (tRNA modification)	4.65e-11	GO:0000049 (tRNA binding)	4.43e-06	GO:0033588 (Elongator holoenzyme complex)	6.92e-20
4	YPR072W YGR134W YDL165W YNR052C YCR093W YER068W YAL021C YNL288W YIL038C	1	GO:0032968 (positive regulation of transcription elongation from RNA polymerase II promoter)	1.62e-21	GO:0004842 (ubiquitin-protein ligase activity)	4.38e-05	GO:0030015 (CCR4-NOT core complex)	1.46e-28
5	YER133W YKL059C YLR115W YGR156W YAL043C YKR002W YJR093C YNL317W YDR301W YDR195W YLR277C YPR107C YNL222W	0.87	GO:0006378 (mRNA polyadenylation)	1.29e-26	GO:0003723 (RNA binding)	1.58e-07	GO:0005847 (CFII complex)	1.20e-37
6	YPL178W YGR013W YIL061C YHR086W YML046W YLR275W YFL017W-A YBR119W YKL012W YLR298C YMR125W YDR240C YPR182W YDL087C YLR147C YDR235W YGR074W	0.81	GO:0000398 (mRNA splicing, via spliceosome)	3.03e-30	GO:0003723 (RNA binding)	5.84e-15	GO:0005685 (U1 snRNP)	3.00e-39
7	YNL166C YJR076C YDR507C YCR002C YHR107C YDL225W YLR314C	–	GO:0000921 (septin ring assembly)	5.30e-15	GO:0005545 (1-phosphatidylinositol binding)	9.62e-10	GO:0000144 (cellular bud neck septin ring)	1.56e-16
8	YHR200W YDL147W YER012W YMR308C YML092C YDL188C YMR314W YMR047C YGL011C YOR362C	–	GO:0010499 (proteasomal ubiquitin-independent protein catabolic process)	2.77e-10	GO:0004298 (threonine-type endopeptidase activity)	3.51e-11	GO:0034515 (proteasome storage granule)	7.89e-12
9	YMR213W YLR117C YPL151C YBR065C YPR182W YLL036C	–	GO:0000398 (mRNA splicing via spliceosome)	4.58e-10	GO:0000384 (first spliceosomal transesterification activity)	5.00e-07	GO:0071006 (U2-type catalytic step 1 spliceosome)	1.85e-06

‘NA’ refers to the neighborhood affinity score between a predicted complex and a reference complex. ‘-‘denotes the NA score is less than 0.2.

## Conclusion

In order to exploit GO to predict protein complexes in a PPI network, we have proposed a novel method which constructs an ontology augmented network based on a PPI network and GO annotation information. Ontology augmented networks can efficiently integrate the PPI data and GO into a unified framework through a unified distance measure. Using the ontology augmented network, we developed a clustering algorithm, COAN, to predict protein complexes, which was capable of taking into account the topological structure of the PPI network, as well as the similarity of GO annotations. Experimental comparisons on two yeast PPI datasets showed that our approach was better than or competitive with the state-of-the-art approaches. In particular, our approach provided a framework to integrate other valuable resources, such as gene expression data.

In a complex, the GO annotations may have different importance. Therefore, they may have a different degree of contribution in the unified distance measure. In future work, we plan to explore a self-adjustment mechanism to determine the degree of contribution of different GO annotations. In addition, we will exploit other resources to improve the performance of COAN in protein complex prediction.

## Supporting Information

Source Code S1 **The source code of COAN.**
(ZIP)
